# A Case of Systemic Lupus Erythematosus With Sole Anti-phosphatidylserine/Prothrombin Complex Antibodies Complicated by Vertebral Artery Dissection

**DOI:** 10.7759/cureus.80437

**Published:** 2025-03-11

**Authors:** Hayato Shimizu, Hiroaki Nishioka

**Affiliations:** 1 Department of General Internal Medicine, Kobe City Medical Center General Hospital, Hyogo, JPN

**Keywords:** anti-phosphatidylserine/prothrombin complex antibodies, antiphospholipid antibodies (apl), systemic lupus erythematosus, vasculopathy, vertebral artery dissection

## Abstract

Cerebrovascular diseases commonly complicate systemic lupus erythematosus (SLE); however, vertebral artery dissection is rare. Although cerebrovascular diseases in SLE are often associated with antiphospholipid antibodies (aPL), such as lupus anticoagulant (LAC), anticardiolipin antibodies (aCL), and anti-β2 glycoprotein-I antibodies, reports of cases with sole positive anti-phosphatidylserine/prothrombin complex antibodies (aPS/PT) are also rare. Herein, we report the case of a 44-year-old woman with SLE who had sole positive aPS/PT results and presented with vertebral artery dissection. The patient, who had previously undergone bypass surgery of the right superficial temporal and middle cerebral arteries for moyamoya vessels, was found to have an asymptomatic left vertebral artery dissection during a follow-up examination. The patient also had a malar rash. Laboratory examination revealed hypocomplementemia and positive results for antinuclear and anti-Smith antibodies. LAC, aCL, and anti-β2 glycoprotein-I antibodies were negative; however, aPS/PT of immunoglobulin G was positive. The patient was diagnosed with SLE with sole positive aPS/PT result complicated by left vertebral artery dissection and moyamoya vessels. We initiated treatment with methylprednisolone pulse therapy, followed by oral prednisolone and intravenous cyclophosphamide. The patient’s condition improved without sequelae. This case suggests that even though patients with SLE presenting with vascular complications lack LAC, aCL, and anti-β2 glycoprotein-I antibodies, other aPLs should be investigated.

## Introduction

Antiphospholipid (aPL) antibodies are defined as antibodies directed against phosphorus-fat components of cell membranes (phospholipids), certain blood proteins that bind with phospholipids, and the complexes formed when proteins and phospholipids bind. Approximately 50% of patients with systemic lupus erythematosus (SLE) produce such antibodies. aPL antibodies are classified into two groups: criteria aPL and non-criteria aPL. Criteria aPL includes lupus anticoagulant (LAC), anticardiolipin (aCL), and anti-β2 glycoprotein-I antibodies and are used in the classification criteria for aPL syndrome (APS) [1,2]. The presence of these antibodies is associated with a predisposition to blood clots. Complications of aPL in SLE include fetal loss and/or miscarriages, blood clots of the veins or arteries, low platelet counts, stroke, Libman-Sacks endocarditis, pulmonary emboli, and spontaneous coronary artery dissection [3-5]. However, in recent years, non-criteria aPL, particularly anti-phosphatidylserine/prothrombin (aPS/PT) complex antibodies, have also been reported as important serological markers for thrombosis and obstetric complications in patients with negative results for all criteria aPL [6]. This report presents the case of a woman with SLE with sole positive aPS/PT results who developed vertebral artery dissection.

## Case presentation

**Figure 1:**
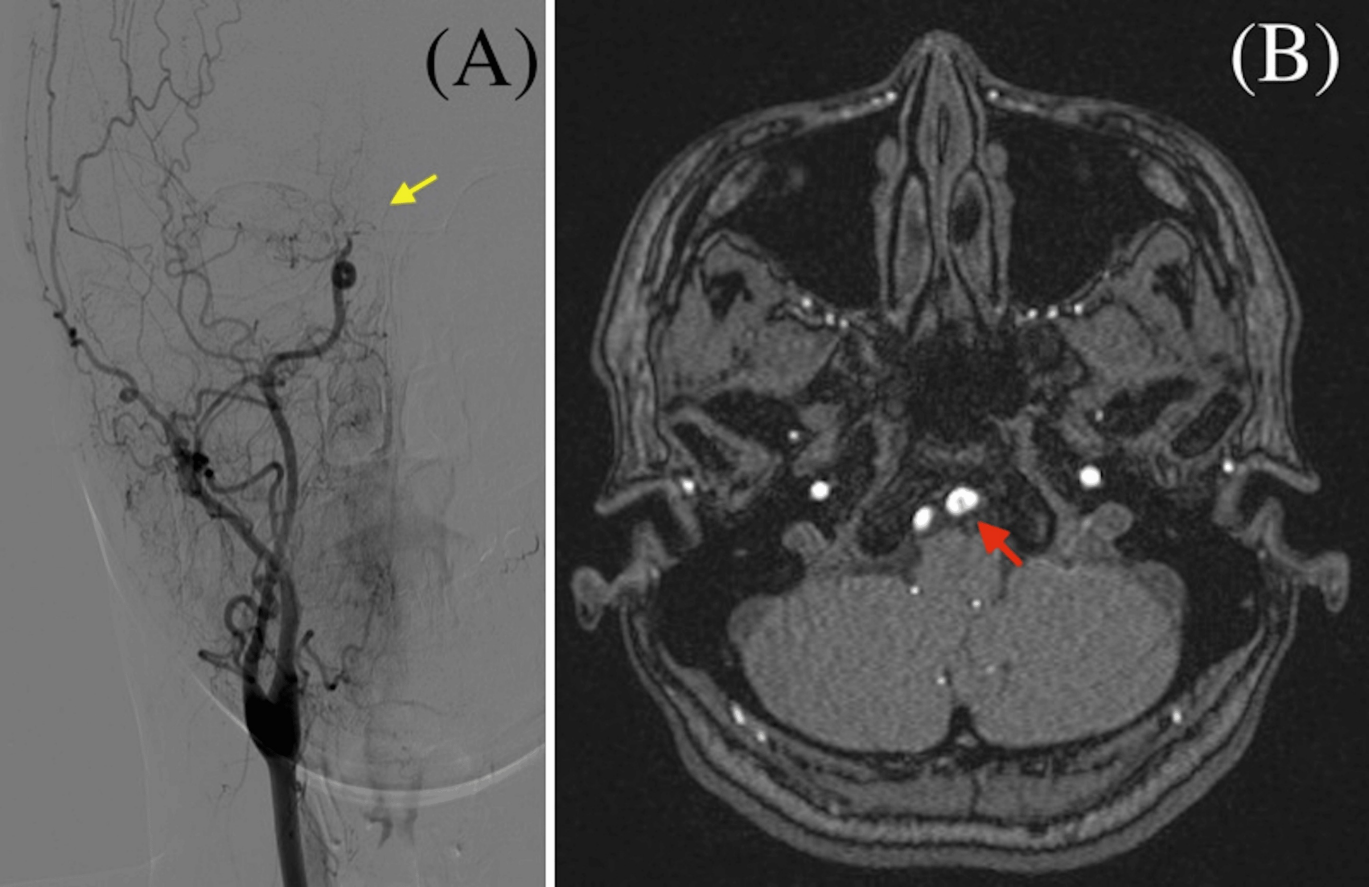
Imaging results of the patient. (A) Brain digital subtraction angiography showing moyamoya vessels and right internal carotid artery occlusion (yellow arrow). (B) Brain magnetic resonance angiography showing left vertebral artery dissection (red arrow).

A 44-year-old woman was admitted to our hospital because of a left vertebral artery dissection incidentally detected by an imaging examination. Seven months earlier, she had developed apraxia and reduced dexterity in her left upper extremity, with moyamoya vessels detected via digital subtraction angiography (DSA) (Figure 1A). Aspirin therapy was initiated, and her symptoms improved. One month later, bypass surgery of the right superficial temporal and middle cerebral arteries was performed. On the day of this admission, the patient visited our outpatient department to undergo a follow-up examination. She was asymptomatic; however, magnetic resonance angiography of the brain revealed left vertebral artery dissection (Figure 1B).

The patient had no recent trauma or special physical activities that stress the cervical arteries. The patient had no history of pregnancy or miscarriage, thrombosis, hypertension, dyslipidemia, or diabetes mellitus, and she had never been a smoker. Physical examination revealed a body temperature of 36.4 ℃, blood pressure of 132/70 mmHg, and pulse rate of 86 beats per minute. The patient was alert and conscious and did not present with neurological deficits. However, she had a malar rash. Laboratory examination revealed mild leukocytopenia and low complement levels. Biochemistry and C-reactive protein levels were normal. Antinuclear antibody was positive, and the level of anti-double-stranded DNA antibody, anti-Smith antibody, anti-U1-ribonucleoprotein antibody, and anti-SSA antibody were elevated. LAC, aCL, and β2-glycoprotein-dependent aCL were negative; however, the aPS/PT of immunoglobulin G (IgG) level was elevated to 15.0 units/mL (cutoff values of 2.0 units/mL) on enzyme-linked immunosorbent assay [7]. Urinalysis revealed no hematuria, proteinuria, or abnormal casts. Cerebrospinal fluid analysis revealed a normal white blood cell count with a mild increase in protein levels (Table 1).

**Table 1:** Laboratory findings on admission. ANA, antinuclear antibody; RNP, ribonucleoprotein; LAC, lupus anticoagulant; aCL, anticardiolipin antibodies; β2GP1, β2 glycoprotein-1; aPS/PT, anti-phosphatidylserine/prothrombin complex antibodies; IL-6, interleukin-6

	Result	Reference range
Blood		
White blood cell (/μL)	3,300	3,900-9,800
Neutrophils (%)	52.4	30-70
Lymphocytes (%)	36.7	19-61
Hemoglobin (g/dL)	10.5	11.1-15.1
Platelet (×10^4^/μL)	25.8	13.0-37.0
Aspartate transaminase (U/L)	34	8-40
Alanine aminotransferase (U/L)	38	8-40
Alkaline phosphatase (U/L)	307	38-113
Lactate dehydrogenase (U/L)	174	124-222
Blood urea nitrogen (mg/dL)	13.7	8.0-20.0
Creatinine (mg/dL)	0.6	0.40-0.80
Na (mEq/L)	139	136-148
K (mEq/L)	3.9	3.5-5.3
C-reactive protein (mg/dL)	0.17	0-0.50
IgG (mg/dL)	2,350	870-1,700
IgA (mg/dL)	233	110-410
IgM (mg/dL)	113	35-220
C3 (mg/dL)	33	65-135
C4 (mg/dL)	5	13-35
ANA	1:360	-
Anti-dsDNA antibody (IU/mL)	90	0-12
Anti-Sm antibody (U/mL)	34.1	0-6.9
Anti-RNP antibody (U/mL)	65	0-12.9
Anti-SSA antibody (U/mL)	127.1	0-9.9
LAC	1.1	0-1.2
aCL (U/mL)	<8.0	0-9.9
β2GP1-dependent aCL (U/mL)	<0.7	0-3.5
aPS/PT (U/mL)	15	0-2
Cerebrospinal fluid		
White blood cell (/μL)	1	0-5
Total protein (mg/dL)	48	15-45
IL-6 (pg/mL)	6.86	Not determined

The patient was subsequently diagnosed with SLE concerning classification criteria [8,9], with sole positive aPS/PT result complicated by left vertebral artery dissection and moyamoya vessels. On day seven, we initiated treatment with intravenous methylprednisolone 1,000 mg/day for three days, followed by oral prednisolone 55 mg/day (1 mg/kg/day) for 14 days, which were subsequently tapered. On day 11, cyclophosphamide 500 mg/m^2^ was administered intravenously, which was repeated monthly. The patient’s C3, C4, and anti-double-stranded DNA antibody levels normalized following treatment initiation. After administering cyclophosphamide nine times, azathioprine and hydroxychloroquine were prescribed, and the prednisolone dose was reduced to 2 mg/day. The patient had no sequelae. To date, the patient has maintained disease remission and has experienced no thromboembolic or vascular events for nine years.

## Discussion

Patients with SLE are often complicated by cerebrovascular diseases, such as stroke, intracerebral hemorrhage, and subarachnoid hemorrhage, which are associated with poor prognosis [10]. Patients with SLE have also been reported to be twice as likely to experience ischemic stroke as individuals without SLE. The causes of ischemic stroke in patients with SLE are primarily attributed to atherosclerosis, APS, vasculitis, or artery dissection [11]. However, vertebral artery dissection in patients with SLE is rare, and only five cases have been reported (Table 2) [12-15]. Among these, two patients showed positive test results for aPL: LAC or aCL [13,14], while aPL was not thoroughly examined in the others. The treatments administered in these reports varied; some patients were managed with antithrombotic drugs alone [13,14], while others received immunosuppressive therapy [13,15]. In addition, papers are reporting an association between moyamoya vessels and SLE [16,17]. We referred to these reports and initiated treatment with intensive immunosuppressive therapy. This case indicates that artery dissection can develop in the vertebral arteries in rare cases and that immunosuppressive therapy may be an option for their treatment in SLE patients with aPL.

**Table 2:** Cases of patients with SLE with vertebral artery dissection. Ref., reference; SLE, systemic lupus erythematosus; LAC, lupus anticoagulant; aCL, anticardiolipin antibodies; NA, not available

Ref.	Age (years), sex	Atherosclerotic vascular risk factor	SLE symptoms	Antiphospholipid antibody status	Treatment
[12]	44 F	None	Mucocutaneous lesions, arthralgia, hemolytic anemia	LAC negative, but otherwise unknown	None
[13]	33 F	None	Nephritis	aCL negative, but otherwise unknown	Antithrombotic therapy
[13]	38 F	Hypertension	NA	aCL positive, but otherwise unknown	Immunosuppressive therapy, antithrombotic therapy
[14]	36 F	None	Nephritis	LAC positive, but otherwise unknown	Antithrombotic therapy
[15]	20 F	None	Fever, thrombocytopenia	unknown	Immunosuppressive therapy, Antithrombotic therapy
Present case	44 F	None	Malar rash, moyamoya vessels, leukocytopenia	aPS/PT	Immunosuppressive therapy, Antithrombotic therapy

The current case is a woman with SLE with sole positive aPS/PT. aPS/PT is one of the most well-known non-criteria aPL. While the positivity rate of aPS/PT of IgG in patients with SLE without APS has been reported to be 13% [7], a sole positive aPS/PT result in patients with SLE, such as in our patient, is rare. aPS/PT positivity usually correlates with the presence of LAC. Positive aPS/PT has a 95%-100% positive predictive value for positive LAC [18], and only 2.3% of patients with autoimmune diseases without LAC and β2-glycoprotein-dependent aCL were reported to have aPS/PT [6].

The relationship between aPL and artery dissection has not been fully established, and further case accumulation of cerebral artery dissection in SLE is required. However, aPL is known to disrupt endothelial cell function, which may be associated with alterations in the balance between vessel wall dilatation and constriction [19], leading to vasculopathy.

In general, common causes of cervical artery dissection include hypertension, trauma, or special physical activities that stress the cervical arteries [20]; however, none of them were observed in our patient. This patient was also noted to have moyamoya vessels. Histological findings of moyamoya vessels have been reported that immune complexes are deposited in the cranial vessels, leading to vasculitis and luminal stenosis or occlusion, suggesting vascular inflammation [17]. In our patient, vascular inflammation may have contributed to vertebral artery dissection, as well as aPS/PT.

## Conclusions

Overall, the present case demonstrates that cases of SLE with sole positive aPS/PT results can be complicated by vertebral artery dissection. A sole positive aPS/PT test result is rare in patients with SLE; however, considering its potential to cause vascular damage, aPS/PT appears to be a clinically meaningful aPL for vascular events in SLE. The presence of aPL may play a role in the development of artery dissection in SLE, and physicians should be mindful of aPL, including non-criteria aPL.
